# Supplementary material for: Non-volatile electrical polarization switching via domain wall release in 3R-MoS2 bilayer
Source: Nat Commun. 2024 Feb 15;15:1389. doi: 10.1038/s41467-024-45709-x (PMC10869714; doi:10.1038/s41467-024-45709-x)
Supplement: Supplementary file 1 — Supplementary Information [file 41467_2024_45709_MOESM1_ESM.pdf]

Non-volatile electrical polarization switching via domain wall release in 3R-MoS_2 bilayer

Dongyang Yang[1,2†], Jing Liang[1,2†], Jingda Wu[1,2†], Yunhuan Xiao[1,2], Jerry I. Dadap[1,2], Kenji Watanabe[3], Takashi Taniguchi[4] and Ziliang Ye[1,2*]

[1]Department of Physics and Astronomy, The University of British Columbia, Vancouver, BC, V6T 1Z1, Canada.
[2]Quantum Matter Institute, The University of British Columbia, Vancouver,BC V6T 1Z4, Canada.
[3]Research Center for Functional Materials, National Institute for Materials Science, 1-1 Namiki, Tsukuba 305-0044, Japan.
[4]International Center for Materials Nanoarchitectonics, National Institute for Materials Science, 1-1 Namiki, Tsukuba 305-0044, Japan.

*E-mail: zlye@phas.ubc.ca;
†These authors contribute equally to this work.

Supplementary Tables and Figures

Based on the extracted peak energy vs. external electric field from Fig.2b, we are able to get the dipole moments of Γ-K excitons (IX_1 and IX_2) and trions (IT_1 and IT_2). The fitting results are listed in the following table (Table 1).

Table 1 μ_1-μ_4 are the dipole moments of IX_1, IX_2, IT_1, and IT_2, respectively. The numbers in the parentheses indicate the uncertainty of the last digit from fitting.

μ_1	μ_2	μ_3	μ_4
0.36(2)	0.40(3)	0.30(2)	0.22(2)

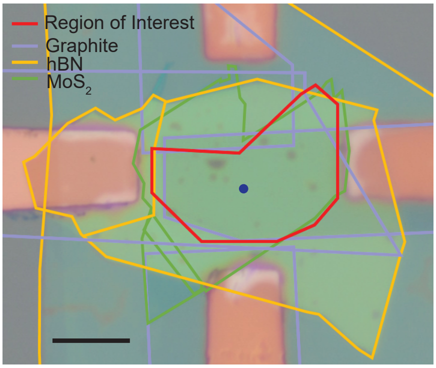

Figure S1: Optical image of Sample 1. The purple, yellow and green curves outline the area of graphite, hBN, and MoS_2, respectively. The red curve outlines the region of interest, where the PL spectrum can be measured. The spot in blue denotes the probing spot for Fig.2 and Fig.3b. Scale Bar: 10 μm

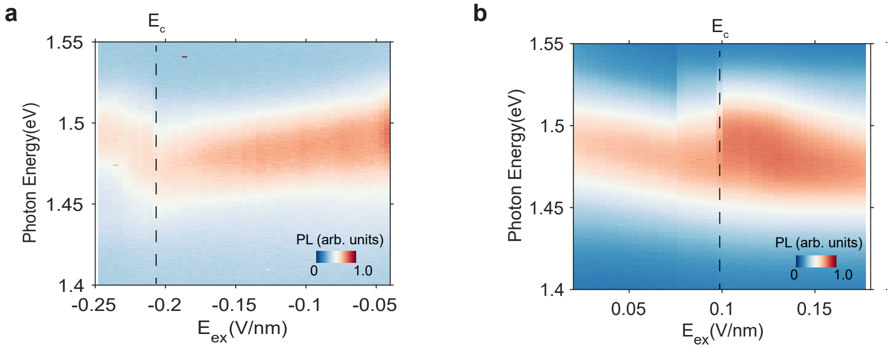

Figure S2: Polarization switching of Sample 2. PL spectra of Γ-K excitons as a function of external electric field (E_{ex}) in sample 2. **a.** E_{ex} is swept towards the negative direction. At the coercive field of E_c=-0.21 V/nm, the domain switches from AB to BA stacking. **b.** E_{ex} scans towards the positive direction. The stacking order switches from BA back to AB at E_c=+0.10 V/nm.

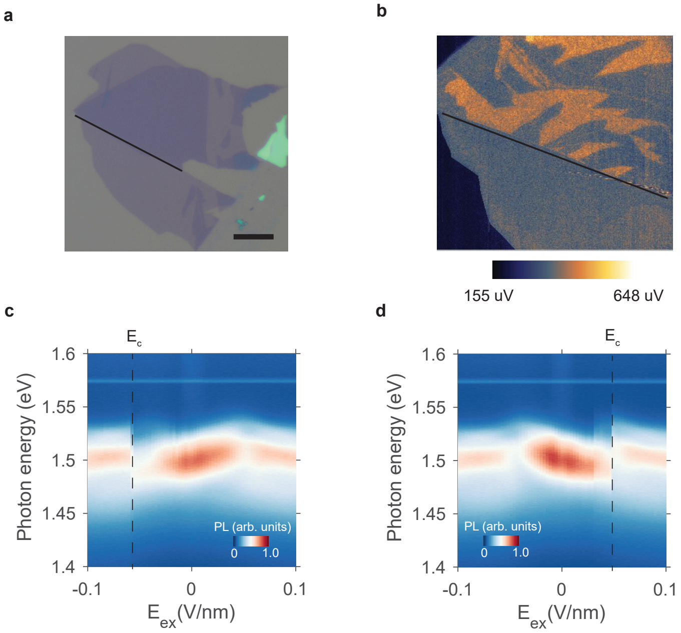

Figure S3: Pre-existing domain walls and polarization switching in Sample 4. a. Optical image of the exfoliated 3R-MoS_2 bilayer in sample 4. Scale bar: 10 μm. **b.** EFM of the flake in (a) prior to its encapsulation. The black lines of **a.** and **b.** indicate the boundary between the regions of monolayer and bilayer. **c.** Photoluminescence spectra of $\Gamma - K$ transitions as a function of the external electric field (E_{ex}). E_{ex} is swept towards the negative direction. At the coercive field E_c=-0.057 V/nm, the stacking order switches from AB to BA at the focus spot. **d.** PL spectra of $\Gamma - K$ transitions versus E_{ex} when the stacking order switches back to AB. The domain switching happens at E_c=0.046 V/nm.

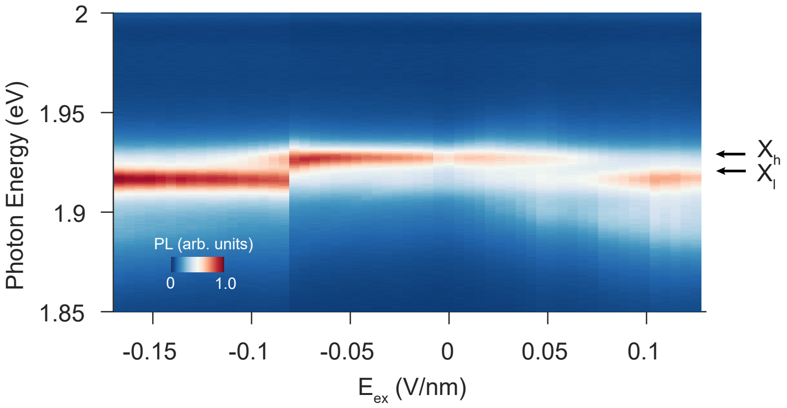

Figure S4: Hysteresis of intralayer excitons. PL spectra of intralayer excitons as a function of external electric field (E_{ex}). X_h and X_l are attributed to the excitons in the top and bottom layers for AB stacking, respectively. E_{ex} is swept towards the negative direction.

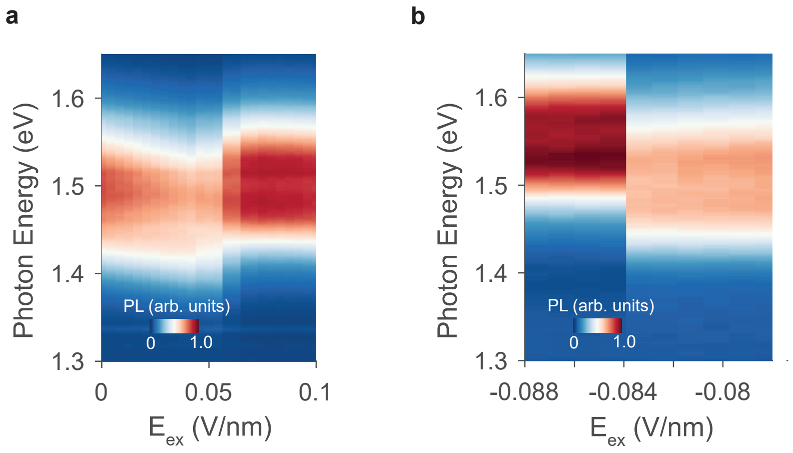

Figure S5: Polarization switching of Sample 1 at room temperature. PL spectra of Γ-K excitons as a function of external electric field (E_{ex}) at room temperature in sample 1. **a.** E_{ex} is swept towards the positive direction. At the coercive field of E_c=+0.063 V/nm, the domain switching from BA to AB stacking. **b.** E_{ex} scans towards the negative direction. The stacking order switches from AB back to BA at E_c=-0.084 V/nm.

Domain switching vs. interface charge trapping effect

The interface charge trapping can be a potential extrinsic contribution to the 2D ferroelectricity phenomena. Experimentally, we observed that the sign of the dipole moment (μ) of Γ-K excitons can be switched by a large training field (Fig.2), indicating the lowest conduction band in the bilayer can be changed from one layer to the other in a non-volatile manner. One possible mechanism behind it is the sliding ferroelectricity effect we describe in the main text: the polarization associated with the stacking order is switched by the training field, and the band alignment between the two layers is reversed by the change in the depolarization field. Nevertheless, there is another possible explanation: Defects in hBN or MoS_2 may become ionized under a large training field, and the ionized charges could get trapped at the interfaces, thus causing a built-in electric field that can also change the band alignment. As discussed below, we can distinguish between these two scenarios by closely examining the intralayer photoluminescence (PL) spectra in Fig.4.

One way to distinguish the trapped charge effect from sliding ferroelectricity is by comparing the intralayer exciton's PL spectra at zero external electric field, after the training of interlayer exciton's dipole moment towards different orientations. In our previous work, we have reported that a bilayer 3R-MoS_2 has an effective type-II band alignment at the K point, where the direct band gaps also have different energies between two layers. In the BA stacking case, where the Mo atom in the top layer is positioned on top of the Sulfide atom in the bottom layer, the band gap in the bottom layer is slightly larger than that in the top layer, corresponding to two intralayer exciton emission peaks near 1.9 eV (X_l and X_h). Moreover, due to the type-II band alignment, the photoexcited electron in the top layer relaxes quickly to the bottom one, causing the low-energy PL peak, X_l, to be weaker than X_h (Fig.S6(a)).

After a large field training, the interlayer exciton's dipole moment is switched. If such a switch is caused by a built-in field from the trapped charge rather than a stacking-order change, we expect that the band gaps in both layers remain the same, and only the conduction band alignment is switched by the built-in field (Fig.S6(b)). As a result, the low-energy peak X_l should become stronger than X_h at zero external field. However, this is not observed in the experiment. As shown in Fig.S6(d), the relative strength of X_l and X_h at zero external field is almost unchanged after training using the largest fields. The high-energy peak is always stronger than the low-energy one. As discussed in the main text, such a switch in the interlayer exciton dipole moment with no change in the intralayer exciton strength can only be explained by a change in the stacking order from BA to AB. In the AB stacking, the top layer has a larger band gap and a lower conduction band (Fig.S6(c)). As a result, the X_h peak remains stronger than X_l, although they are emitted from different layers after the switch.

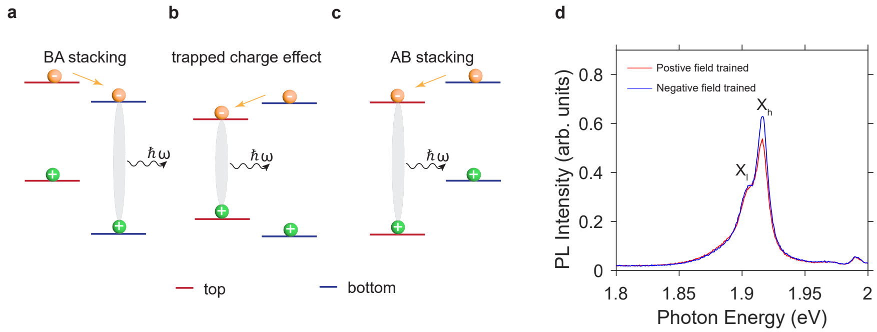

Figure S6: Domain switching vs. interface charge trapping a. Schematic of the band alignment for a BA-stacked 3R-MoS_2 bilayer. **b.** Schematic of the conduction band inversion under the built-in field induced by trapped charges at the interface. **c.** Schematic of the band structure after the stacking order is changed from BA to AB. **d.** PL spectra of intralayer excitons in sample 1 measured at zero external electric field. Red: The sample is trained by a large positive field, indicating the AB stacking domain. Blue: The sample is trained by a large negative field and the domain, corresponding to the BA stacking domain.

Estimation on the Joule heating effect

In this section, we provide an estimation on the temperature increase of 3R-MoS_2 bilayer induced by delocalized electrons during the propagation of the domain wall.

We first assume a 10x10 μm square 3R-MoS_2 bilayer (Fig.S7) with a domain wall initially pinned at the left edge. The length of the domain wall L_{DW} is therefore 10 μm and its width is negligible compared to the flake size. Under an external electric field, the domain wall is released from the pinning center and moves towards right. We estimate the domain wall propagation speed v to be on the same order as the speed of sound in MoS_2, approximately 10^4 m/s.[1]. The time interval Δt for the domain switch to finish is therefore about 1 ns. Within such a short time, thermal conduction has not finished and the process is approximately adiabatic. Thus, the instantaneous temperature increase ΔT can be estimated using the equation:

$$C\Delta T = IU\Delta t \tag{1}$$

Here $C\approx 10^{-13}$ J/K is the heat capacity of this 10x10 μm 3R-MoS_2 bilayer, estimated according to **ref.2**. The right side of the equation (1) is the Joule heating induced by the delocalized charges. I is the current when electrons

transfer from one layer to another layer (Equation (2)).

$$I = \frac{\Delta q}{\Delta t} = \frac{2Pv\Delta t L_{DW}}{\Delta t} = 2PvL_{DW} \quad (2)$$

Here **P** is the polarization, which is 0.55 μC/cm^2. U is the total interlayer potential when the external electric field is close to the coercive field E_c (Equation (3)).

$$U = \phi + E_c \times d_0 \quad (3)$$

The depolarization field induced interlayer potential ϕ is 58 mV. The coercive field E_c is around 0.07 V/nm in the sample 1. $d_0 \approx 0.70$ nm is the interlayer distance. Thus, the total interlayer potential is about 0.1 V at the switching point.

Based on the equations (1)-(3), the estimated temperature increase due to Joule heating is about 1 K. Since we do not observe any obvious temperature dependence from 4 K to 300 K, we do not expect that such Joule heating caused by the interlayer transfer of delocalized electrons can significantly affect the domain wall motion.

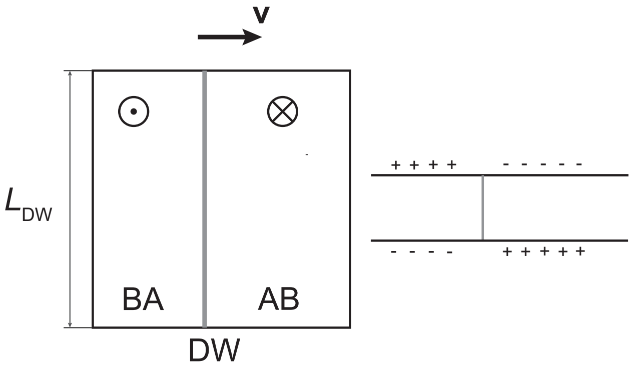

Figure S7: Schematic of an ideal model for the domain switching. Top and side views of the polarization switching in a 3R-MoS_2 bilayer. The simplified flake is a 10x10 μm square. Domain wall (grey) is moving towards the right edge.

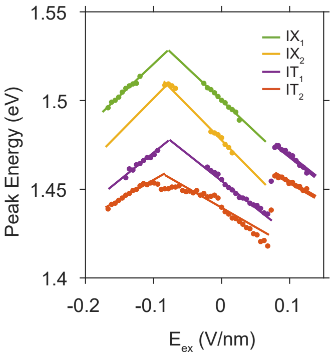

Figure S8: Additional data on the exciton Stark shift. a. The peak energy as a function of E_{ex} when E_{ex} is scanned towards the positive direction. IX_1, IX_2, IT_1, and IT_2 are labeled by green, yellow, purple, and red dots. The solid lines serve as guidelines.

References

1. Cong Xin, et al. Probing the acoustic phonon dispersion and sound velocity of graphene by Raman spectroscopy. *Carbon* **149**, 19-24 (2019)

2. Saha Dipankar, and Santanu Mahapatra. Analytical insight into the lattice thermal conductivity and heat capacity of monolayer MoS2. *Physica E: Low-dimensional Systems and Nanostructures* **83**, 455-460 (2016)
